# In Situ Transfer of Laser‐Induced Graphene Electronics for Multifunctional Smart Windows

**DOI:** 10.1002/smsc.202400010

**Published:** 2024-06-21

**Authors:** Tongmei Jing, Han Ku Nam, Dongwook Yang, Younggeun Lee, Rongke Gao, Hongki Yoo, Soongeun Kwon, Seung‐Woo Kim, Liandong Yu, Young‐Jin Kim

**Affiliations:** ^1^ College of Control Science and Engineering China University of Petroleum (East China) Qingdao 266555 China; ^2^ Department of Mechanical Engineering Korea Advanced Institute of Science and Technology (KAIST) Science Town Daejeon 34141 South Korea; ^3^ Nano‐Convergence Manufacturing Systems Research Division Korea Institute of Machinery & Materials (KIMM) Daejeon 34103 South Korea

**Keywords:** direct laser writings, glass electronics, in situ transfers, laser‐induced graphenes, multifunctional windows

## Abstract

The ascent of internet of things (IoT) technology has increased the demand for glass electronics. However, the production of glass electronics necessitates complicated processes, including conductive materials coating and chemical vapor deposition, which entail the use of additional chemicals. Consequently, this raises environmental apprehensions concerning chemical and electronic waste. In this study, a fast, cost‐effective, and simple approach are presented to meet the growing demand for glass electronics while addressing environmental concerns associated with their production processes. The method involves converting polyimide (PI) tape into laser‐induced graphene (LIG) and transferring it onto a glass substrate using ultraviolet laser direct writing technology. This process allows for the fabrication of LIG‐embedded glass without additional chemical treatments in ambient air. Subsequently, the residual PI tape is removed, resulting in LIG‐based glass electrodes with an electrical resistivity of 1.065 × 10^−3^ Ω m. These LIG electrodes demonstrate efficient functionality for window applications such as defogging, heating, temperature sensing, and solar warming, suitable for automotive and residential windows. The potential scalability of this eco‐friendly technology to IoT‐based smart and sustainable window electronics further underscores its adaptability to meet diverse user needs.

## Graphical abstract

Herein, the UV laser direct writing and in situ transfer of laser‐induced graphene (LIG) are demonstrated on glass to form key electrical components for smart windows applications. These LIG electrodes demonstrate efficient functionality for window applications such as defogging, heating, temperature sensing, and solar warming, suitable for automotive and residential windows.

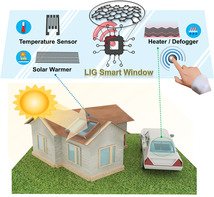

## 1 Introduction

Since the inception of the term internet of things (IoT) in 2002, continual advancements have driven the evolution of technologies facilitating remote control over interconnected devices.^[^ ^1^ ^]^ IoT fundamentally enables smart connectivity and management of machinery or tools via the internet and sensors.^[^ ^2^ ^]^ To actively extend IoT technology, endeavors have concentrated not only on the integration of control tools such as sensors but also on the incorporation of conductive materials, such as conductive ink, into nonconductive substrates.^[^ ^3^ ^]^ This empowers these objects to function as sensors or antennas themselves. However, amid growing environmental concerns and the increasing importance of eco‐friendly electronics, there is a rising demand for technologies and materials that minimize environmental impact.^[^ ^4^ ^]^ While the specific legal and social regulations for green electronics remain somewhat unclear, Mihai Irimia‐Vladu outlined four crucial requisites in 2014: abundant, inexpensive organic materials as precursors; economically viable synthetic routes with high production rates; cost‐effective fabrication processes for synthesized electronic materials; and biodegradable, biocompatible properties in the green device.^[^ ^5^ ^]^ Therefore, the application of this technology in enabling object conductivity for IoT must align with eco‐conscious practices. Essentially, amid the expanding landscape of IoT‐based technology, its future scalability significantly hinges on the concurrent fulfillment of green electronics criteria in implementing object conductivity.

Glass, an inorganic material cooled down to a solid state without crystallization, attracts constant attention as the ideal material for implementing IoT due to its optical transparency, structural rigidity, compositional flexibility, tailored property suitability, and durability.^[^ ^6^ ^]^ Utilized since its ancient origins around 2000–3000 BC, glass has found application in a diverse range of items—from cups, dishes, and car windows to modern‐day laptop monitors and smartphones.^[^ ^7^ ^]^ Glass electronics are progressively advancing, particularly amid the current technological advancements. Diverse technologies are developing for displays and electronics, enabling the production of semiconductor devices on amorphous substrates like glass.^[^ ^8^ ^]^ This innovation effectively curtails substrate expenses while preserving transparency. Moreover, the emergence of smart glass, incorporating an electrochromic film within the glass to alter optical properties based on voltage, has gained traction across various applications.^[^ ^9^ ^]^ Furthermore, efforts to infuse conductivity into glass substrates involve methodologies such as ITO electrode coating,^[^ ^10^ ^]^ high‐temperature synthesis of single‐crystal metal nanowires,^[^ ^8^ ^]^ advancement in metallic glass manufacturing,^[^ ^11^ ^]^ and the creation of metal nano‐trough fibers via electrospinning.^[^ ^12^ ^]^ Continuous advancements in deposition methods further facilitate the implementation of transparent glass electronics. In addition, the rising interest in graphene, known for its eco‐friendliness, low resistivity, thermal stability, and exceptional transmittance, has fueled the steady development of glass electronics leveraging graphene and carbon‐based materials. Various carbon deposition techniques, including thermal chemical vapor deposition (CVD)^[^ ^13^ ^]^ and plasma‐enhanced CVD for graphene application,^[^ ^14^ ^]^ alongside methods like spin‐coating for graphene oxide (GO)^[^ ^15^ ^]^ and carbon nanotube deposition through CVD,^[^ ^16^ ^]^ have been reported for fabricating glass electrodes. However, these electrode formation processes often involve extensive time and cost due to the application of conductive materials across the entire glass, coupled with additional steps like etching, thereby posing challenges to their eco‐friendliness in electronics production.

The laser‐induced graphene (LIG) formation technology, which converts carbon materials into eco‐friendly electrodes through laser direct writing (LDW), has gained consistent attention since its inception. Introduced by the James Tour group in 2014 for generating LIG on polyimide (PI),^[^ ^17^ ^]^ this method has expanded to various substrates such as polymers,^[^ ^18^ ^]^ wood,^[^ ^19^ ^]^ leaves,^[^ ^20^, ^21^ ^]^ fabrics,^[^ ^22^, ^23^ ^]^ and paper.^[^ ^24^ ^]^ The resulting LIG electrodes have found applications in an array of bending and strain sensors,^[^ ^25^, ^26^ ^]^ chemical^[^ ^27^ ^]^ and biosensors,^[^ ^28^ ^]^ temperature^[^ ^29^ ^]^ and gas sensors,^[^ ^30^ ^]^ water purification filters,^[^ ^31^ ^]^ heater,^[^ ^32^ ^]^ supercapacitors,^[^ ^33^ ^]^ triboelectric nanogenerator,^[^ ^34^ ^]^ and more. Despite its diverse utility, the widespread application of LIG on glass remains relatively unexplored at present. In 2019, a study explored transferring LIG electrodes onto glass via hot pressing.^[^ ^35^ ^]^ However, this method necessitates additional complex procedures post‐LIG formation, posing limitations. Subsequently, in 2022, research focused on developing reduced GO electrodes by coating GO on glass,^[^ ^36^ ^]^ yet this process involves complex GO deposition on PET and discarding PET remnants post‐transfer. Another 2022 technology employed laser‐induced backward transfer to create and transfer LIG in real time, using PI between two glasses to suppress ablation.^[^ ^37^ ^]^ Nevertheless, in this scenario, the use of two glass pieces may lead to material contamination and subsequent waste, contributing to the ongoing limitations of this technology. The presented studies highlight extensive technologies on LIG, yet there remains a notable scarcity of research focusing specifically on glass. Even within the limited studies available, unresolved issues persist, indicating the need for further investigation and overcoming existing challenges.

Herein, we developed a technology using nanosecond UV‐LDW to create LIG and in situ transfer it onto glass. Through irradiating a commercial PI tape attached to the glass with a high‐photon‐energy UV wavelength laser pulse, the PI tape transforms into LIG. Simultaneously, the strong laser pulse induces a partial decomposition and recombination process on the glass surface, resulting in LIG–glass composites. The technology described herein shares similarities with laser‐induced forward transfer (LIFT), a method of transferring material from a donor to a receiver.^[^ ^38^ ^]^ In this instance, the donor comprises a PI tape, while the receiver may consist of a glass substrate. Although LIFT technology has undergone extensive study and optimization, our approach differs in that it eliminates the need for an additional donor substrate. Instead, the PI tape deposited on glass is converted to LIG through photon energy and simultaneously embedded. This approach offers the advantage of enhanced convenience and simplicity. Under optimal conditions, we successfully created LIG electrodes on glass with an electrical resistivity of 1.065 × 10^−3^ Ω m. We developed a technology aiming to integrate electronics into transparent windows. Our approach involves implementing a defogger, a temperature sensor, and a solar warmer, seamlessly integrating them through UV‐LDW‐based LIG electrodes. This technology offers versatile configurations of electronics without the need for complex installation or additional processing, allowing quick, simple, and customizable manufacturing to suit individual needs. Notably, it utilizes carbon electrodes, an eco‐friendly material, without adding additional chemicals, addressing waste issues inherent in electronics and enabling eco‐friendly recycling. This innovative approach marks a step forward in sustainable glass electronics, offering a swift and eco‐friendly route to implement IoT‐based smart windows.

## 2 Results and Discussion

### 2.1 In Situ Transfer of LIG Electrodes on Transparent Glass

**Figure 1:**
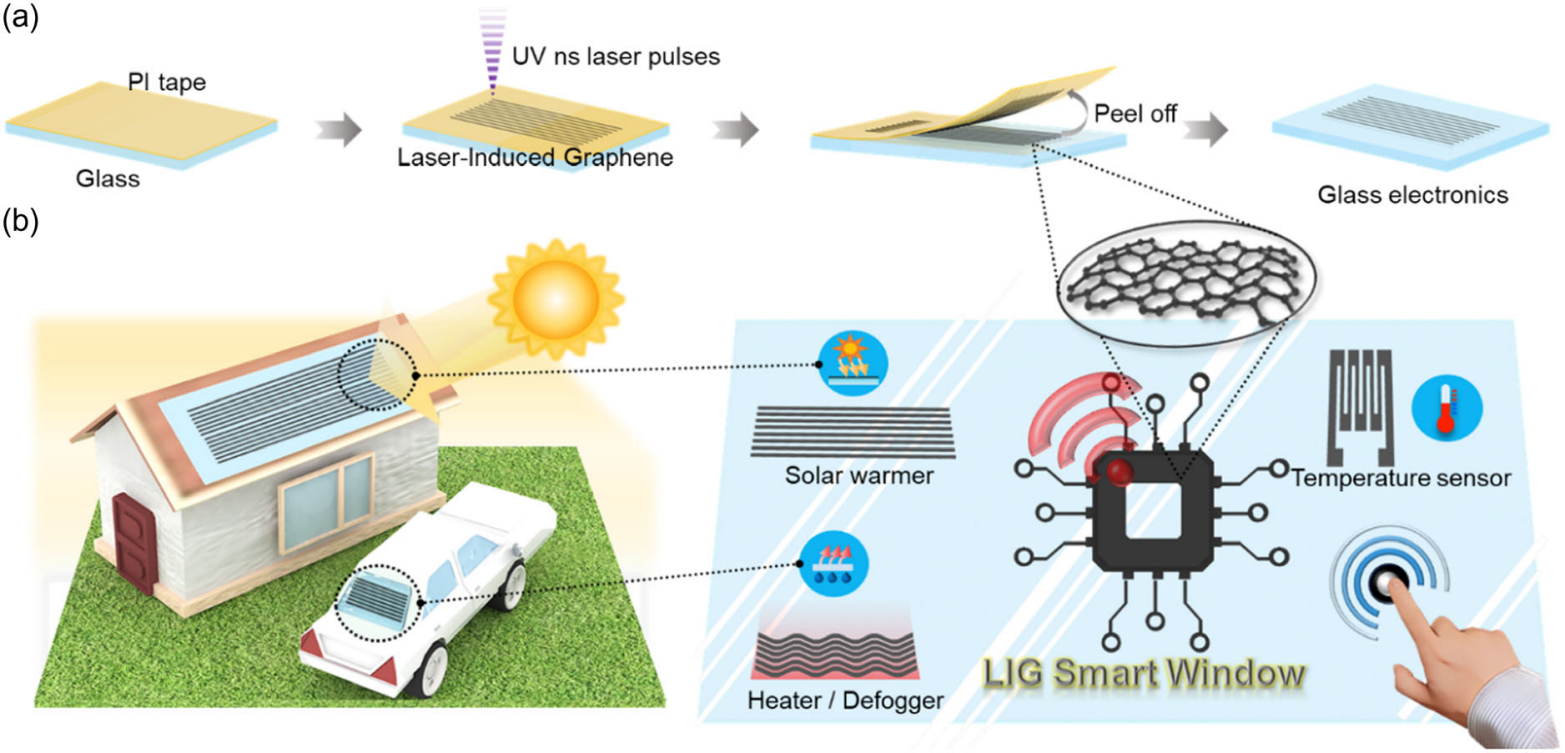
Schematic and concept image of LIG‐embedded glass for smart window applications. a) Schematic diagram of in situ transfer of LIG electrodes to the glass substrate. b) Concept image of LIG‐based solar warmer, a temperature sensor, and a defogger for smart window applications.

In this research, we fabricated LIG by employing PI tape, subsequently transferred to glass as seen in **Figure** **1**. This technique facilitated the swift and facile production of glass electronics while enabling the integration of smart window functionalities such as solar absorption‐based warming, defogging, and temperature sensing. Our approach underscores the practicality of devising an eco‐friendly electrode on nonconductive glass without intricate processes. Illustrated in Figure 1a, applying a nanosecond 357 nm UV laser to a PI tape‐attached soda‐lime glass induces carbonization and graphitization, transforming the PI into LIG. Specifically, the laser's photon energy breaks C=O, C—O, and N—C bonds, liberating molecules into gas.^[^ ^39^ ^]^ The ensuing high gas pressure curbs the decomposition of carbon precursors, fostering the formation of small carbon clusters that amalgamate into larger graphene structures.^[^ ^40^ ^]^ The liberated gas minimizes oxidation during the conversion to graphene, while the accumulated thermal energy partially melts the glass surface, encapsulating the generated LIG. This process yields LIG–glass composites, where subsequent removal of the non‐irradiated PI tape results in glass electronics embedded with LIG electrodes. As seen in Table S1, Supporting Information, the investigation conducted LIG formation on glass with a 10 mm length across different laser scanning speeds (4.0–30 mm s^−1^) and laser powers (ranging from 1.0 to 11 W). Ultimately, under the parameters of 8.0 W and 10 mm s^−1^, the optimal resistivity value condition of 1.065 × 10^−3^ Ω m was attained. Although certain conditions produced lower resistivity of LIG, these conditions lacked adherence to the glass and were prone to easy removal. This discrepancy likely arises from the energy disparity required for glass ablation versus high‐quality LIG formation from PI. Especially, the high absorption rate of UV photons primarily facilitates PI to LIG transformation on the surface. The investigation of PI‐based LIG transformation on glass substrates involves understanding the thermal dynamics of the conversion process. Specifically, the process utilizes precise thermal control where the temperature is sufficient to initiate the carbonization and graphitization of PI (typically occurring between 2400 and 3000 K) while simultaneously approaching the melting point of the glass substrate, which is around 1400 °C for soda‐lime glass.^[^ ^39^, ^41^, ^42^ ^]^ This careful utilization of temperature control converts PI to LIG, while the glass substrate begins to soften without fully melting, creating a conducive environment for the newly formed LIG to embed into the glass surface. The localized heating effect of the laser not only transforms the PI into LIG but also slightly softens the glass surface. This softening (around 1850 K) allows the LIG to become embedded into the top layer of the glass, creating a seamless interface between the two materials.^[^ ^43^, ^44^, ^45^, ^46^ ^]^ This embedding is crucial for the integration of LIG into the glass, facilitating a robust mechanical interlocking that enhances the durability and utility of the composite material. In our study, temperature simulations played a pivotal role in understanding the thermal dynamics involved in the formation of LIG on glass substrates. The simulations revealed a base temperature of ≈1000 K and a peak temperature reaching up to 3000 K during the laser processing (details are described in Figure S1, Supporting Information).^[^ ^47^, ^48^ ^]^ These temperatures are critical for the transformation process, where the base temperature closely approaches the melting point of soda‐lime glass, and the peak temperature is well within the range required for the carbonization and graphitization of PI to form LIG. Also, thermal profile ensures that the glass substrate softens to a point where the newly formed LIG can embed into its surface without causing full melting or structural compromise. The embedding of LIG into the slightly softened glass surface is facilitated by the high peak temperature, which ensures the formation of a robust mechanical interlock between the LIG and the glass, enhancing the composite's durability and functionality. Utilizing the LIG‐embedded glass generated through this process, we've introduced functional enhancements, including an LIG solar warmer to augment indoor temperatures via solar absorption, and an LIG temperature sensor (Figure 1b). Additionally, we've devised an LIG heater‐based defogger and defroster capable of eliminating moisture and frost, culminating in the realization of smart window technology.

### 2.2 Characterization of Embedded LIG on Glass

**Figure 2:**
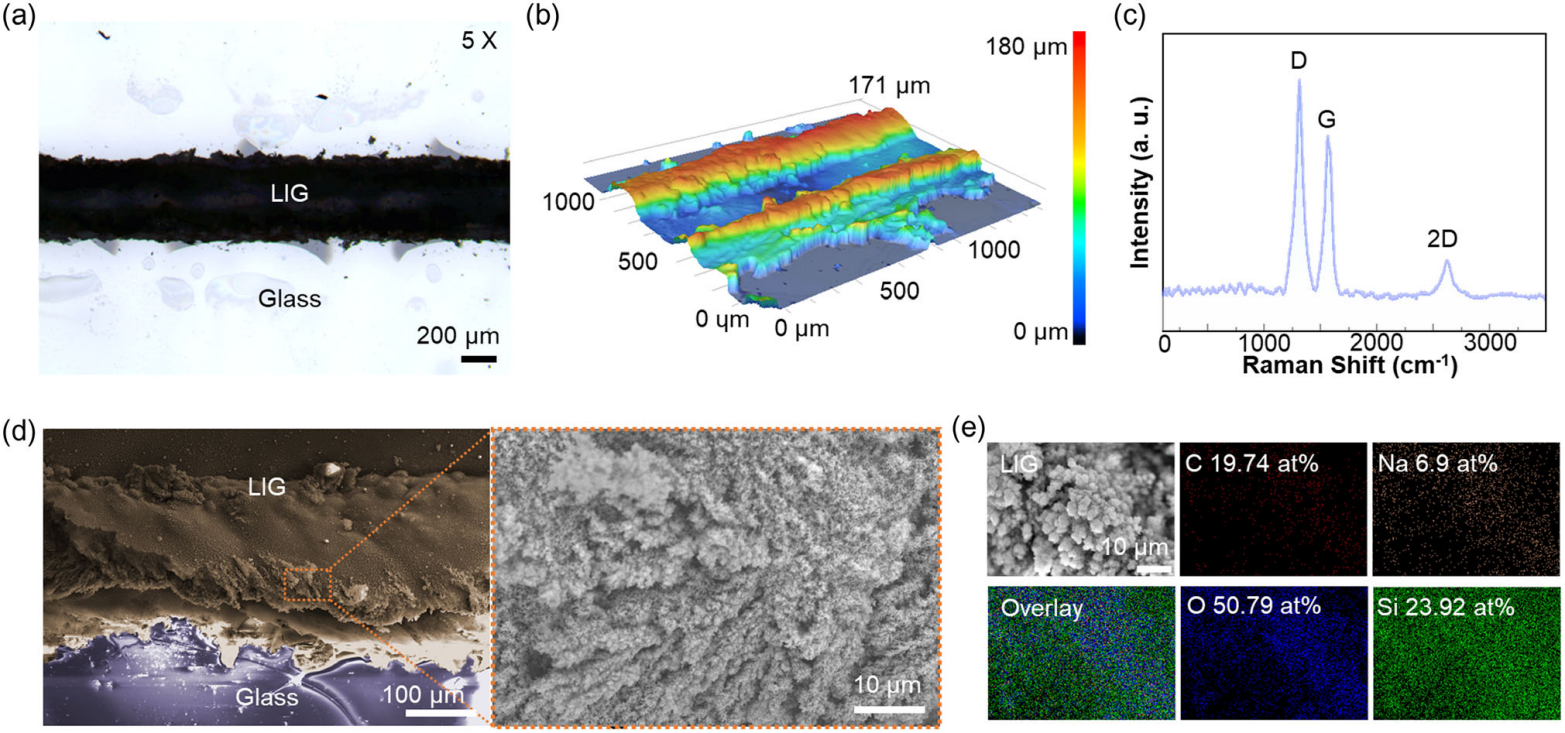
Mechanical and chemical characterizations of LIG on glass. a) Optical microscopy image of LIG‐embedded glass. b) Confocal microscopy of the LIG‐embedded glass. c) Raman spectrum of LIG‐embedded glass. d) SEM image of glass and LIG‐embedded glass. e) EDS images of LIG‐embedded glass.

The findings depicted in **Figure** **2** validate the consistent performance of the produced LIG electrodes on glass, as evaluated through an analysis of their physical and chemical properties. Under the optimal condition of 1.065 × 10^−3^ Ω m, examination via an optical microscope revealed a uniform formation of LIG–glass composites, showcasing a width of 467 μm without any observable defects as seen in Figure 2a. The process involves the excitation of electrons in the PI surface by the absorbed laser pulse energy, initiating subsequent electron–phonon interactions that transfer energy to the lattice. This elevated lattice temperature facilitates the transformation process from PI to graphene, resulting in the deposition of LIG on the glass surface.^[^ ^49^, ^50^ ^]^ This concurrent enhancement in the absorption rate of incident laser energy enables precise local laser ablation on the glass surface, forming the LIG–glass composite.^[^ ^51^ ^]^ Under the laser condition of 8.0 W with 6 mm s^−1^ as seen in Figure S2, Supporting Information, the slower writing speed led to excessive heat accumulation owing to the strong laser pulse energy during the conversion of PI to LIG. Consequently, this overextended the glass ablation area, causing damage. Simultaneously, the line width of LIG widened to 695 μm, and the resistivity is 4.008 × 10^−3^ Ω m, marking four times increase compared to the optimal condition. Conversely, insufficient energy was applied at a high speed of 20 mm s^−1^, resulting in the localized generation of LIG only at the center, leading to a line width of 315 μm. The markedly increased resistivity of 3.408 × 10^−1^ Ω m means poor conversion of PI to LIG, with most electrodes being disconnected. For the conversion of PI into high‐quality LIG, specific conditions are indispensable—a high temperature between 2400 and 3000 K and a pressure of 3.2 GPa by outgassing resulting from temperatures ranging from instantaneous decomposition via pulse energy. Deviations from this temperature or pressure range, either higher or lower, disrupt the reassembly of disassembled carbon sources into the desired ring structure or cause the collapse of the polymer backbone, resulting in incomplete generation of LIG, primarily as amorphous carbon.^[^ ^40^, ^42^ ^]^ In essence, achieving LIG with lower resistivity necessitates the precise calibration of laser power and scanning speed. Furthermore, despite the beam spot size being ≈80 μm, the linewidth resolution of graphene under optimal conditions spans several hundred micrometers (467 μm), as adequate heat energy is necessary for the transformation of PI into LIG and its subsequent embedding into glass. To achieve a finer line width for an LIG electrode, one approach entails further reducing the beam size by opting for an f‐θ lens or an objective lens with a shorter focal length and depth of focus. To investigate the thickness variation of the produced LIG relative to glass thickness, 1 and 0.15 mm thick glass substrates were coated with 0.25 mm PI tape and patterned using optimal laser conditions (8 W, 10 mm s^−1^), as depicted in Figure S3, Supporting Information. Subsequently, the thickness of the resulting LIG–glass composites was assessed via optical microscopy, as illustrated in Figure S4, Supporting Information. Due to the porous structure and uneven thickness of LIG, the average measurement from several points was considered representative. The LIG formed on 1 mm thick glass exhibited a thickness of 0.640 mm, while on 0.15 mm thick glass, it measured 0.253 mm. This indicates an LIG–glass composite formation rate of 64% and 169% relative to the glass substrate, respectively. The photothermal energy generated during laser irradiation decomposes PI and glass, enabling their simultaneous recombination into deep LIG–glass composites. Particularly, the occurrence of porous structure expansion during LIG generation contributes to the ratio exceeding 100% for thinner glass. We conducted three tests for each glass thickness (as detailed in Table S2, Supporting Information) to assess the mass loss during the transformation of PI tape to LIG. Laser‐induced thermal decomposition causes both the PI tape and glass to undergo weight reduction, albeit the LIG–glass composites are heavier than glass due to melting and subsequent solidification, ensuring effective encapsulation of LIG. Interestingly, the ratio of mass loss varied with glass thickness, while the difference in mass loss remained ≈0.04 g regardless of thickness. This suggests that under consistent energy input, PI–glass undergoes a proportional conversion to LIG–glass composites, with negligible influence from the glass substrate on LIG production conditions, provided it maintains structural integrity.

The investigation utilizes confocal microscopy analysis to compare LIG and glass substrates under optimized laser processing conditions of 8.0 W power and 10 mm s^−1^ scanning speed, presenting the results in Figure 2b, S5, Supporting Information. Initially, a disparity in height of 27–34 μm emerges between the center of the LIG–glass composite and the glass substrate. This contrast stems from the Gaussian laser shape, concentrating high photon energy at the center, leading to significant removal of the PI tape. Only a residual portion remains after carbonization and graphitization of PI tape, interacting with the glass and resulting in a relatively minor height difference. Conversely, the disparity in height, ranging from 138 to 142 μm, between the glass substrate and the LIG–glass composite near its edge occurs due to significant energy in the center by laser, propelling decomposed gases outward. The graph at the bottom of Figure S5, Supporting Information displays the cross‐section profiles of the LIG–glass composites produced, illustrating the height variations among the center, edge, and glass substrate. Consequently, the carbon rings resulting from graphitization reassemble with the molten glass, forming a composite exhibiting relatively high conductivity, combining the state of LIG with glass. Additionally, the measurement of average roughness for the center and edge LIG–glass composites indicates a distinct pattern: the line edge (*R*_a_edge_) registers at 5 μm, while the line center (*R*_a_center_) records 11 μm. A silicon micro/nanostructure within the LIG center contributes to heightened surface roughness, while the resolidification process of LIG–glass is anticipated to diminish roughness by integrating the glass into the porous LIG structure.

Figure 2c illustrates the outcomes of the Raman spectroscopy analysis conducted on LIG–glass composites. The Raman spectrum of LIG glass reveals prominent peaks at 1320 and 1569 cm^−1^, corresponding to the characteristic D and G peaks associated with carbon‐based structures. The D peak emanates from defects in the sp^2^ carbon atoms within the aromatic ring, signifying disordered graphene. Concurrently, the G peak correlates with the in‐plane vibrational mode of carbon atom pairs within the graphene framework.^[^ ^52^ ^]^ Moreover, the presence of a 2D band at 2627 cm^−1^ indicates secondary domain boundary phonons, validating the formation of a graphene structure.^[^ ^53^, ^54^ ^]^ The relatively heightened D peak, in contrast to optimal LIG in previous studies, stems from our LIG electrodes being embedded within glass and partially integrated with Si, a byproduct of molten glass. The details were further revealed through scanning electron microscopy (SEM) and energy‐dispersive spectroscopy (EDS) analyses, showcased in Figure 2d,e. The SEM images in Figure 2d delineate a nanoscale porous structure on the outer periphery of LIG–glass composites. This structure is notably visible even at lower magnifications (Figure S6a, Supporting Information). It can be formed by the outward extension of gas resulting from the dissociation of the PI tape by photon energy, originating from the center. Conversely, while the center appears relatively flat at lower magnifications, a closer inspection reveals relatively uniform nanostructures (Figure S6b, Supporting Information). The evidence confirms that the amorphous state of glass undergoes crystallization through a decomposition–recombination process during the formation of LIG.^[^ ^55^ ^]^ Throughout this transformation process, carbon and silicon are extracted from the glass and combine. Elemental analysis via EDS comparing LIG–glass composites (Figure 2e) and glass (Figure S7, Supporting Information) showcases the presence of SiO_2_ as fused silica, constituting 21.3% of silicon and 60.3% oxygen initially. Post‐laser irradiation, Si increases to 23.9%, accompanied by a decrease in oxygen ratio to 50.8%. Meanwhile, carbon, the primary component of LIG, escalates from 2.73% to 19.74%, suggesting the elimination of most suspended substances including oxygen during laser irradiation, with decomposed Si assisting in carbon assembly. Notably, Si‐based adhesive from the PI tape might contribute to the reassembly process. Through a medium capable of directly absorbing the laser's photon energy, like PI atop the glass, energy is transferred to the glass by means of direct phonon transport and inelastic electron scattering at the PI–glass interface.^[^ ^56^ ^]^ This process results in the transformation of PI into LIG while concurrently inducing the conversion of amorphous glass into polycrystalline Si.

### 2.3 Demonstration of LIG Heater on Glass for Defogger Application

**Figure 3:**
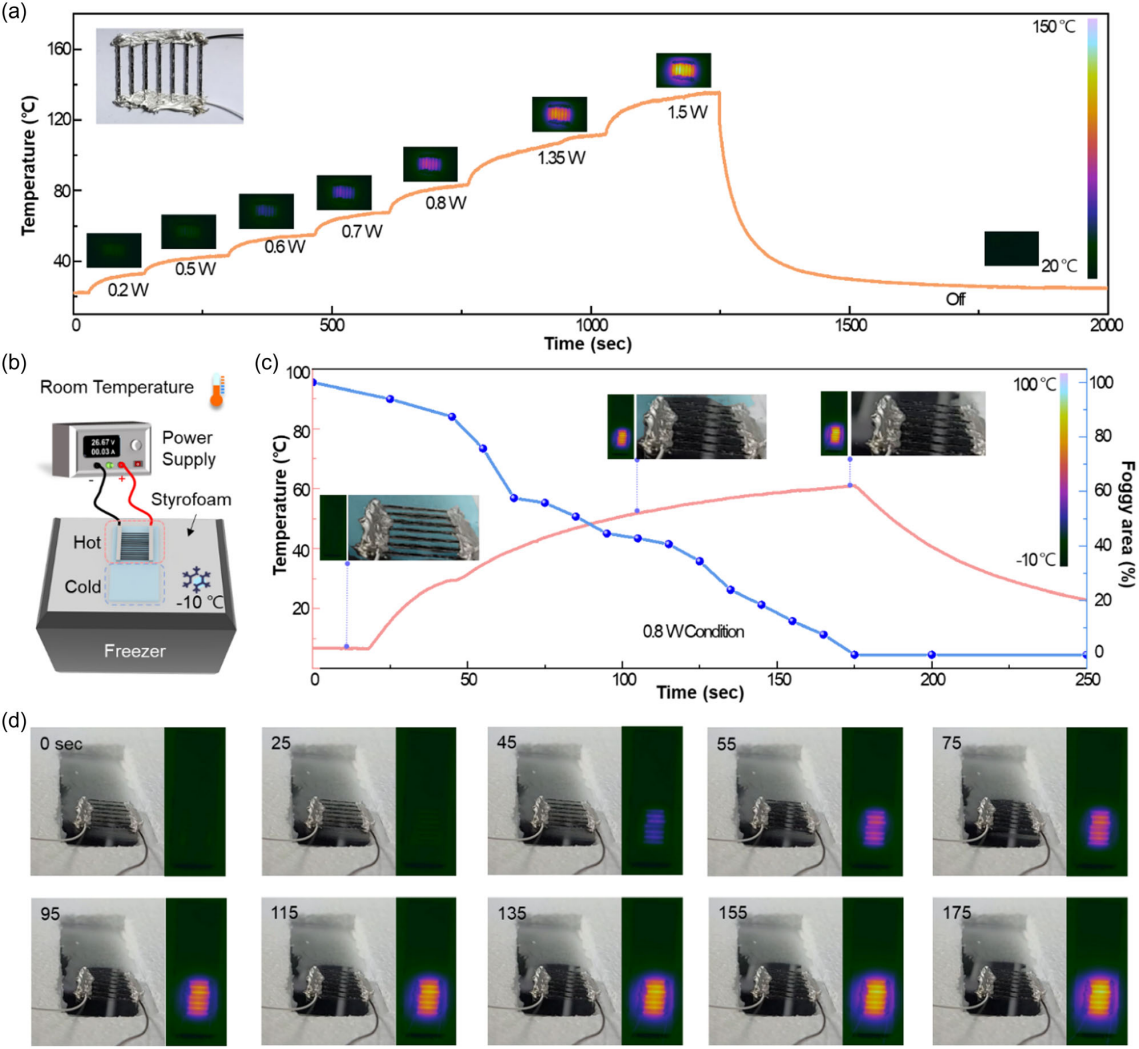
Characterization of the LIG heater on glass. a) Temperature profile of an LIG heater patterned on glass with incremental increases in applied power from 0.2 to 1.5 W and the corresponding infrared (IR) images. b) Defogging test system. c) Defogging performance evaluation with LIG heater operating at 0.8 W. d) Real‐time photographs and the corresponding IR images of LIG heater and bare glass.

The incorporation of a conductive circuit, embedding LIG electrodes on glass, holds promise for transparent heaters with a wide array of applications. These heaters operate based on the Joule heating effect, where the electrical resistance of LIG electrodes transforms electrical energy into thermal energy. The study demonstrates the integration of the LIG heater onto glass, showcasing heating, and efficient defogging capabilities, as illustrated in **Figure** **3**. Applying power under varied voltage conditions results in a rapid surface temperature rise of the LIG heater, reaching normal levels within a few minutes as seen in Figure 3a. At 1.5 W (50 V, 0.03 A), the maximum temperature attained is 135.6 °C, remaining stable without fluctuations. Following the cessation of power, the temperature decreases from its maximum to room temperature within 8 min, stabilizing at 33.36 °C, which corresponds to a T_90_ stabilization time of 153 s. Further, an antifog test highlights the utilization of the LIG heater as seen in Figure 3b and S8, Supporting Information. In a winter‐simulated environment (−10 °C) by using a freezer, creating a temperature contrast with room temperature, the glass surfaces fogged. The antifog performance was evaluated by positioning an LIG heater on one glass surface and placing another bare glass next to the LIG‐heater‐installed glass. Leveraging Joule heating, the electric heat defogger swiftly eliminates fog. As seen in Figure 3c, monitoring the LIG heater's temperature variation during defogging at 0.8 W (40 V, 0.02 A) reveals a gradual temperature increase reaching a saturation point at ≈150 s, subsequently stabilizing. Concurrently, as the heater's temperature rises, the foggy area diminishes rapidly, with about 50% cleared within 75 s and complete clearance achieved once the heater temperature saturates within 175 s as presented in Figure 3d and S9, Supporting Information. The defogging results underscore the remarkable potential of LIG heaters on glass for facile, rapid, and cost‐effective deployment in transparent electric antifog devices, particularly in realms such as vehicle windows, outdoor displays, and insulated windows.

### 2.4 Demonstration of LIG Temperature Sensor and 1D Grid for Energy Saving

**Figure 4:**
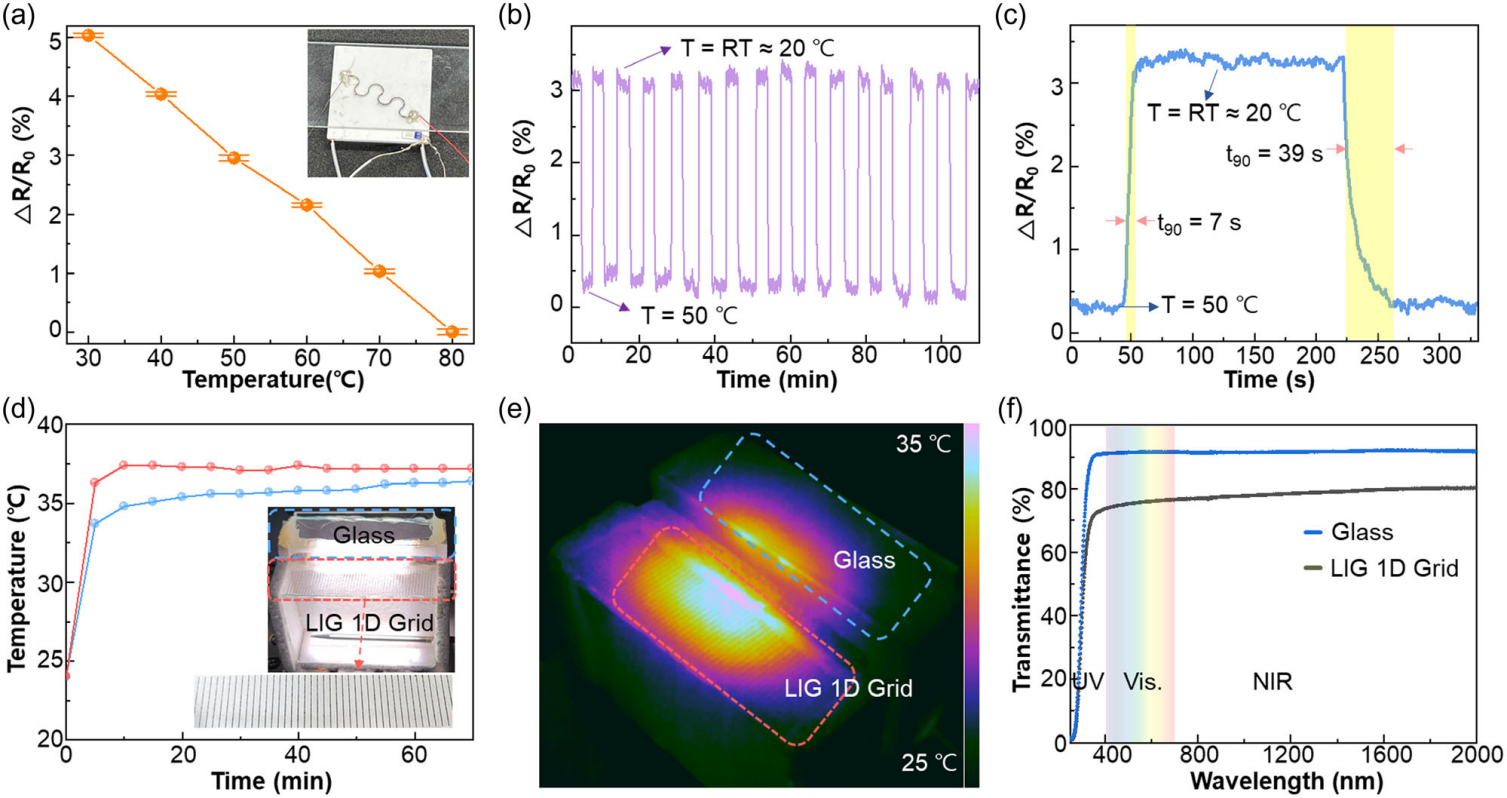
a) Variations of LIG temperature sensor's electrical resistance within the temperature ranges of 30–80 °C. b) Repeatability test of LIG temperature sensor. c) Time responses of the LIG temperature sensor between the room temperature (20 °C) and 50 °C. d) Real‐time temperature monitoring of LIG 1D grid (red line) and glass (blue line) under solar simulator 1 sun illumination. e) Thermal image of the LIG 1D grid and glass under 1 sun illumination. f) UV–vis transmission spectrum of the LIG 1D grid and glass.

Creating a temperature sensor using LIG on glass aimed to enable advanced monitoring for smart windows, showcased in **Figure** **4**a–c. Employing in‐situ transfer techniques, LIG was precisely patterned onto the glass substrate to fashion a predesigned temperature sensor. A comprehensive dataset was generated by systematically altering temperatures from 30 to 80 °C, calculating average electrical resistance at each interval, elucidated in Figure S10, Supporting Information. The resulting graph exhibits a negative temperature coefficient of resistance (TCR) of ≈−0.1% °C^−1^, as presented in Figure 4a. TCR is calculated by following the equation.

(1) $$\text{TCR }\left(\%\right)=\frac{\Delta R}{R_{0}\cdot \Delta T}\times 100$$

Notably, the TCR of this temperature sensor showcases commendable quality compared to other graphene‐based sensors, surpassing values such as LIG derived from wood (−0.025% °C^−1^),^[^ ^32^ ^]^ LIG from Kevlar (−0.097% °C^−1^),^[^ ^23^ ^]^ LIG from paper (−0.028% °C^−1^),^[^ ^57^ ^]^ and graphene synthesized via the CVD process (−0.0017% °C^−1^).^[^ ^58^ ^]^ Additionally, evaluating the response and recovery times involved subjecting the sensor to stepwise temperature changes between 20 °C (representing room temperature) and 50 °C, detailed in Figure 4b,c. The negative TCR observed in LIG‐based temperature sensors can be attributed to the unique properties of LIG and the mechanisms that govern its electrical conductivity at different temperatures.^[^ ^57^, ^59^, ^60^ ^]^ In general, the TCR of a material indicates how its electrical resistance changes with temperature. For most metals, the TCR is positive, meaning that their resistance increases with temperature due to increased phonon scattering.^[^ ^61^, ^62^ ^]^ However, for some materials like graphene, the TCR can be negative, which means their resistance decreases as temperature increases. The negative TCR in LIG‐based temperature sensors is primarily due to the semimetallic nature of graphene. Graphene's charge carriers (electrons and holes) increase in mobility as the temperature rises, leading to a decrease in resistance. This behavior can be explained by several mechanisms, including thermally activated hopping, band structure modification of graphene, and phonon scattering of graphene's unique lattice structure.^[^ ^63^, ^64^, ^65^ ^]^ In thermally activated hopping, electrons within a solid exhibit the phenomenon of hopping, where they overcome energy barriers to move to adjacent locations. This behavior becomes more pronounced with increasing temperature, particularly prevalent in semimetals like graphene. Band structure deformation involves alterations in the energy band structure due to external factors such as pressure and temperature, leading to changes in resistance. For graphene, elevating temperature results in a narrowing of the energy bandgap, correlating with a decrease in resistance. Phonon scattering involves the vibration of phonons, primarily induced by temperature changes in the crystal lattice. As temperature rises, atoms vibrate, causing phonon propagation and collisions with electrons. In metals, this impedes electron mobility, thus increasing resistance. Conversely, graphene's distinctive energy band structure tends to mitigate resistance, resulting in a propensity for decreased resistance under temperature variation. Remarkably, the response and recovery times measured at 7 and 39 s, respectively, showcase excellent performance suitable for smart window applications. These timings are influenced by factors such as the glass thickness (1 mm) and the thermal conductivity of the glass (0.5 W (m K)^−1^).

The LIG 1D grid solar warmer, designed for solar thermal heating devices showcased in Figure 4d–f, was evaluated using an experimental setup detailed in Figure S11, Supporting Information. The solar simulator generated an intensity equivalent to 1 sun, directed into two model rooms. One of the top windows was characterized by the presence of the LIG 1D grid on glass (with the other walls composed of glass and foam), while the control windows featured bare glass. Figure 4d demonstrates that the room temperature reached ≈37.2 °C with the LIG 1D grid solar‐warmer‐installed room, slightly surpassing the temperature of the bare‐glass‐installed room at around 36.3 °C, showing an increase of over 1 °C. Notably, the LIG 1D grid solar‐warmer‐installed room rapidly achieved equilibrium within 10 min, significantly faster than the bare‐glass‐installed room, which required roughly 60 min. This demonstrates the LIG 1D grid‐based glass's ability to swiftly absorb solar heat and maintain a stable temperature compared to bare glass. In Figure 4e, simultaneous temperature monitoring further validates this superior performance in thermal imaging, affirming stability and high efficiency in solar absorption and thermal conductivity. Given that a 1°C reduction in rising temperature can lead to energy savings of 7–14%, the LIG 1D grid solar warmer shows the potential to maintain a temperature over 1°C higher solely using solar energy, positioning it as an outstanding energy‐saving solution.^[^ ^66^ ^]^ Additionally, the transmittance analysis between the LIG 1D grid solar warmer and exposed glass, as displayed in Figure 4f, reveals a 20% reduction in light transmittance compared to bare glass. This data underscores the efficient absorption of natural light by the LIG 1D grid for subsequent conversion into heat. Moreover, exhibiting over 70% transmittance in both the visible light and IR bands, alongside considerations for absorption and scattering by the 1D grid, solidifies its suitability as a glass window. The performance of LIG electronics on glass, specifically the LIG 1D grid solar warmer, capable of functioning as a heater and temperature sensor, displays immediate applicability for installation in homes and cars, as depicted in Figure S12, Supporting Information. This innovation holds promise as a noteworthy solar heating device and a potential smart, eco‐friendly window alternative.

## 3 Conclusion

This study presents a method wherein the PI tape is attached to a glass substrate and converted into LIG using UV‐LDW technology, embedding it into the glass simultaneously. Upon removing the PI tape from the non‐laser‐irradiated area, the remaining area secures transparency except the LIG‐embedded area, enabling the implementation of glass electronics. Employing a UV nanosecond laser with high absorption, along with the single‐step transfer of carbon electrodes to glass in ambient air, without necessitating special chemical treatment, enhances the attractiveness of this approach. Optimization led to the acquisition of the resistivity of 1.065 × 10^−3^ Ω m of 30 mm long LIG electrodes, further extended into IoT‐driven smart window technologies. The LIG heater‐embedded glass reaches temperatures up to 135 °C via Joule heating, resolving real‐life issues such as defogging and defrosting in automobiles and house windows. Significantly, defogging tests revealed the complete removal of fog within 3 min, highlighting its commercial potential. The LIG electrode also serves as a reliable temperature sensor with a high TCR coefficient of −0.1% °C^−1^ and robust stability across repeated tests, suggesting real‐time temperature measurement for windows and glass. Additionally, the LIG‐based 1D grid solar warmer effectively elevates indoor temperatures by over 1 °C, offering temperature stability superior to regular glass, potentially saving more than 7% energy for heating while maintaining high transmittance of over 70% across a wide wavelength band. This technology stands as a next‐generation, energy‐saving solution applicable to both automotive and residential window systems. These technologies provide an affordable, rapid, and customizable means to implement window electronics, catering to diverse consumer needs and potentially expanding into various IoT‐based glass electronics. Moreover, the eco‐friendly nature of this approach, without the use of additional chemicals, offers a sustainable solution and addresses the recycling E‐waste problem associated with window electronics.

## 4 Experimental Section

4.1

4.1.1

##### LIG on Glass: Patterning Details

The commercial PI tape was prepared for in situ transfer of LIG electrodes onto soda‐lime glass (VWR, 631‐1160) measuring 76 × 25 mm with 1 mm thickness and another soda‐lime glass (Matsunami, S9213) measuring 76 × 52 mm with 1.3 mm thickness. Following the manufacturing process of LIG, which involved irradiating a nanosecond UV laser while attaching PI tape onto glass without any specific treatment for in situ transferring of LIG electrodes onto the glass, the residual tape was subsequently removed. The UV‐LDW setup, elaborated in Figure S13, Supporting Information, comprised diverse optical components to precisely direct the UV laser's beam onto the sample, integrating a galvano scanner and f‐θ lens. With 11 ns pulse duration, 357 nm ultraviolet wavelength (Figure S14, Supporting Information), 11 kHz repetition rate, and lasers of up to 20 W (JPT, Seal‐355‐20S), the initial‐stage LIG electrodes were produced directly in the ambient atmosphere without any supplemental chemical processes. UV laser pulses, managed by optical elements like a quarter‐wave plate, half‐wave plate, and polarized beam splitter, facilitated precise control over polarization and output parameters. Subsequently, the lasers were navigated using the galvano scanner equipped with computer‐programmable mirrors as per the designated design. To achieve high‐resolution patterning, the beam was focused through an F‐θ lens (SL‐355‐60‐100). The galvano mirror system scanned the laser beam, after being focused by an f‐θ lens onto the sample, produced a spot size of 80 μm with a focal length of 100 mm (Figure S15, Supporting Information). The optimization process involved systematic adjustments in laser output (ranging from 1.0 to 11 W) and scan rate (modulated between 4 and 30 mm s^−1^). All experimental procedures were conducted under atmospheric conditions (20–25 °C, relative humidity 35–55%) without additional chemical treatments. The resistance was measured five times using a conventional multimeter, and the average value was documented. For calculating the resistivity of LIG electrodes as seen in Table S1, Supporting Information, an optical microscope was used to measure the height and width of the LIG electrode. The resistivity (ρ) of the LIG electrode was calculated by following the equation.

(2) $$\rho =\frac{RA}{L}$$

where *R* represents resistance of the LIG electrode, *A* denotes the cross‐sectional area of the electrode, and *L* signifies the length of the electrode. This process confirmed the stable formation of optimized, high‐quality LIG electrodes on a glass substrate under the specified conditions of 8.0 W laser power and 10 mm s^−1^ scanning speed.

##### Material Characterization

The surface of LIG–glass composites was scanned by optical microscope (OLYMPUS BX53M) with 5× objective lens. Raman spectroscopy was performed in HORIBA LabRAM ARAMIS dispersive spectrometer equipped with a 50× objective lens. Measurements were performed with a 633 nm laser with 10 s exposure time and five accumulations as measurement parameters, to capture G, D, and 2D Raman peaks. The ablation depth and average roughness of LIG–glass composites were assessed utilizing a confocal microscope (Keyence, VK‐X200). The morphology and chemical composition were also studied through EDS mounted in an SEM (FEI Company, Magellan 400). As seen in Figure 2, the morphology and chemical composition were observed using SEM (FEI Company, Magellan 400) combined with EDS working at an accelerating high voltage of 20 kV with current of 0.80 nA of the electron beam, horizontal field width (HFW) of 635 μm, work distance of 4.5 mm, and the Everhart–Thornley detector in magnified condition of 500×, and working at an accelerating high voltage of 10 kV with current of 0.40 nA of the electron beam, HFW of 63.5 μm, work distance of 4.5 mm, and the through‐the‐lens detector in magnified condition of 2000×.

##### Smart Windows Applications Performance Test

LIG obtained from PI tape was selectively transferred in a predesigned pattern through the utilization of a UV nanosecond laser operating at an average power of 8 W and a scanning rate of 10 mm s^−1^. The LIG heater and LIG temperature sensor was connected with silver paste‐connected wires at both ends. Activation of the heater was achieved using an electrical power supply (Keysight, U8032A). Real‐time temperature monitoring was conducted with an IR camera (FLIR, A35). For defogging testing, a cold environment simulation system was manufactured (Figure S8, Supporting Information), which was equipped with freezer (Joy Tutus, CR22) covered with styrofoam. The temperature of the freezer was set to −10 °C, creating a temperature differential with the room temperature, resulting in the formation of fog on the sample surface. Temperature control was achieved using a ceramic heater (Thorlabs, model HT24S) and regulated by a temperature controller (Thorlabs, model TC200). Temperature measurements were monitored by a Pt thermistor, specifically the TH100PT model from Thorlabs. LIG temperature sensor's resistance value was in situ monitored by precision source/measurement unit (Keysight, B2912A). The performance of the LIG 1D grid solar warmer was evaluated under sunlight intensity equivalent to 1 sun, generated by a solar simulator (ORIEL, LCS‐100TM). Real‐time temperature monitoring was performed using an IR camera (FLIR, A35). The temperatures inside the model rooms were monitored using two electronic thermometers. The transmission spectrum was acquired using Fourier transform infrared spectroscopy (Thermo Scientific, Nicolet iS50).

## Conflict of Interest

The authors declare no conflict of interest.

## Author Contributions

T.J., H.K.N., and D.Y. equally contributed to this work. Y.‐J.K., H.K.N., L.Y., T.J., D.Y., and S.‐W.K. conceived the idea. T.J., H.K.N., and D.Y. designed the experiments and prepared the manuscript. T.J., H.K.N., and D.Y. carried out heater and sensor experiments. T.J., H.K.N., D.Y., and Y.L. performed sample fabrication. T.J. H.K.N., D.Y., R.G., H.Y., and S.K. conducted characterizations. Y.‐J.K., H.K.N., and L.Y. supervised all the experiments. All the authors contributed significant discussions for final article polishing.

## Supporting information

Supplementary Material

## Data Availability

The data that support the findings of this study are available from the corresponding author upon reasonable request.
