# Supplementary material for: In Situ Transfer of Laser‐Induced Graphene Electronics for Multifunctional Smart Windows
Source: Small Sci. 2024 Jun 21;4(9):2400010. doi: 10.1002/smsc.202400010 (PMC11934981; doi:10.1002/smsc.202400010)
Supplement: Supplementary file 1 — Supplementary Material [file SMSC-4-2400010-s001.pdf]

[Supporting Information]

In-situ Transfer of Laser-Induced Graphene Electronics for Multifunctional Smart Windows

Tongmei Jing [1,2,†], *Han Ku Nam* [2,†,*], *Dongwook Yang* [2,†], *Younggeun Lee* [2], *Rongke Gao* [1], *Hongki Yoo* [2], *Soongeun Kwon* [3], *Seung-Woo Kim* [2], *Liandong Yu* [1,*], *and Young-Jin Kim* [2,*]

T. Jing, Prof. R. Gao, Prof. L. Yu
College of Control Science and Engineering, China University of Petroleum (East China), Qingdao, 266555, China
E-mail: liandongyu@upc.edu.cn

T. Jing, Dr. H. K. Nam, D. Yang, Y. Lee, Prof. S.-W. Kim, Prof. Y.-J. Kim
Department of Mechanical Engineering, Korea Advanced Institute of Science and Technology (KAIST), Science Town, Daejeon, 34141, South Korea
E-mail: hanku.nam@kaist.ac.kr
E-mail: yj.kim@kaist.ac.kr

Dr. S. Kwon
Nano-Convergence Manufacturing Systems, Research Division, Korea Institute of Machinery & Materials (KIMM), Daejeon 34103, South Korea

[Temperature Evolution under Laser Pulses]

Energy Deposition and Temperature Rise

The deposition of energy from femtosecond laser pulses is closely linked to the energy on the surface according to the fluence of the laser pulse. The energy density induced on the surface by heat, referred to as P, from a pulse at a specific point (xc, yc) is given by an equation where α signifies the portion of energy retained on the surface, Ep is the pulse's energy, and w0 is the width of the laser beam.

$$\mathbf{P} = \frac{\boldsymbol{\alpha} \cdot \boldsymbol{E_P}}{\boldsymbol{\pi} \cdot \boldsymbol{w_0}} \boldsymbol{e}^{-\frac{2((x-x_c)^2+(y-y_c)^2)}{w_0{}^2}}$$

The rise in temperature due to a spike in surface energy is calculated with this formula:

$$\boldsymbol{T_{rise}} = \frac{\boldsymbol{E}}{\boldsymbol{c} \cdot \boldsymbol{\rho} \cdot \Delta \boldsymbol{x} \Delta \boldsymbol{y} \Delta \boldsymbol{z}}$$

In this formula, T_{rise} is the temperature rise, E is the energy introduced, inversely related to the material's density (ρ), its specific heat capacity (c), and the volume of an element (ΔxΔyΔz). In our calculations, the parameter α is defined as the energy absorption rate (%), signifying the portion of incident laser energy retained on the surface of the material. This definition aligns with the conventional understanding in laser-material interaction studies, where α represents the surface energy absorption efficiency. The energy absorption occurs strictly on the surface, and for polyimide (PI) at 357 nm, the absorption rate is almost 100%, meaning α = 1.[1,2] Skin depth refers to the depth at which the laser intensity falls to 1/e of its original value at the surface. For polyimide at 357 nm, the skin depth is relatively low (inversely proportional to absorption rate), further supporting the assumption that energy absorption predominantly occurs at the surface.[3] The skin depth implies that volume absorption is minimal and does not significantly affect the surface modification process.

Heat Dispersion Over Time

Over time, the initial temperature increase dissipates as heat spreads throughout the material. This diffusion is described by the heat conduction equation, which relates changes in temperature over time and space, with ρ representing density, c the specific heat, and k the thermal conductivity. For solving the heat equation, certain assumptions are required. The entire equation is addressed at the stationary point, with the laser moving at a constant speed. For the rising temperature graph, it is assumed that there is no heat loss to the surroundings. Similarly, for the relaxation graph, no external heat input is considered.

$$\rho \cdot c \cdot \frac{\partial T}{\partial t} = \frac{\partial}{\partial x}\left(k \cdot \frac{\partial T}{\partial x}\right) + \frac{\partial}{\partial y}\left(k \cdot \frac{\partial T}{\partial y}\right) + \frac{\partial}{\partial z}\left(k \cdot \frac{\partial T}{\partial z}\right)$$

Then, the heat dissipation equation is simplified into following equation. (k: thermal conductivity, c: heat capacity, L: effective length).

$$\mathrm{T}(0,\mathrm{t}) = T_0 + (T_{max} - T_0)e^{-\beta t}, \beta = \mathrm{k}/(\rho c L^2)$$

According to the transient Temperature rise that discussed in last section and heat dispersion equation, the thermal behavior of PI under repetitive pulsed laser irradiation can be calculated.

Thermal Behavior under Repetitive-Pulse Laser

In processes involving repetitive-pulse lasers on a PI tape/glass surface, the energy retained at a particular surface point is determined by the laser's pulse repetition rate and the scanning speed. The scanning speed affects the accumulated energy, while the pulse repetition rate decides the heat spread between pulses. A high frequency is necessary to get the base and peak temperatures. The time-temperature graph is obtained with multiple pulses at a 110 kHz repetition rate, 8 W average power, a 10 mm/s scan speed, and a laser focus centered at x = -40 μm (half the beam's width), show a base temperature of 1000 K and a peak of 3000 K. These conditions were modeled considering the properties of Polyimide (Density: 1430 kg/m³, Thermal conductivity: 0.24 W/(m·K), Initial temperature: 300 K). Every calculation is solved by MATLAB software.

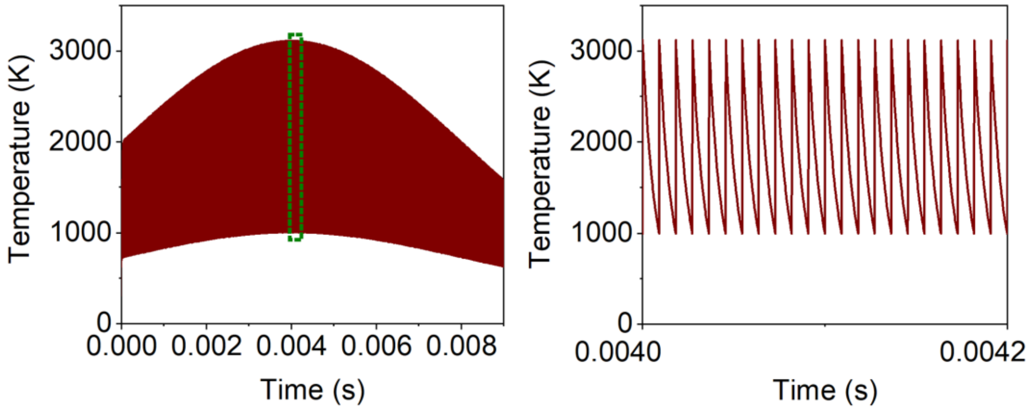

Figure S1. Temperature evolution during the nanosecond pulse irradiation on PI tape/glass.

Table S1. Resistivity values of LIG-glass composites with different power and laser scanning speed.

Resistivity (Ω*m × 10^{-3})						
W \ mm/s	4	6	8	10	20	30
1	579.8	-	-	-	-	-
2	242.5	-	-	-	-	-
3	505.7	358.6	1193	-	-	-
4	62.39	54.07	957.1	385.9	-	-
5	2.855	50.80	7.166	19.36	-	-
6	4.683	25.49	4.104	10.05	-	-
7	22.45	20.31	1.988	1.665	-	-
8	64.72	4.008	1.556	**1.065**	621.3	-
9	1.846	7.271	4.526	1.719	430.8	-
10	15.00	3.268	50.96	217.3	217.1	-
11	17.61	7.986	47.11	44.14	137.6	-

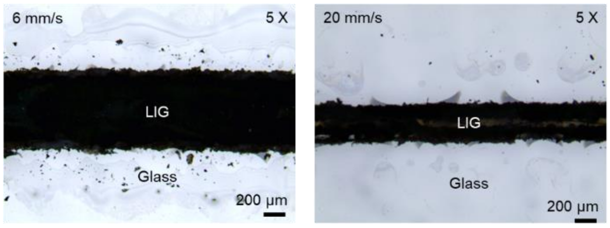

Figure S2. Microscope images of LIG-glass composites line with different laser scanning speeds.

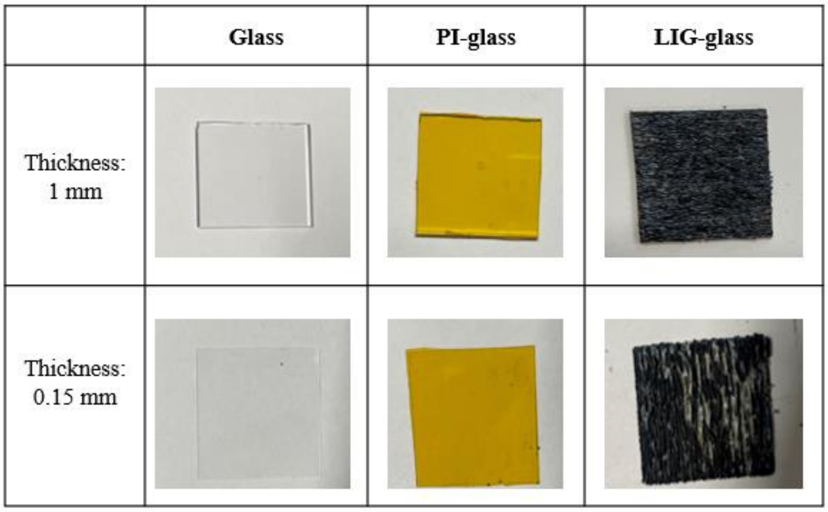

Figure S3. LIG-glass composites with different thickness of glass substrates.

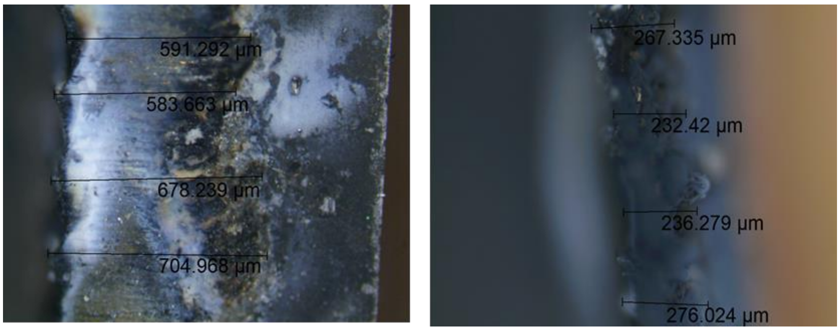

Figure S4. Microscope images of LIG-glass composites thickness with different glass. (left) 1 mm thickness of glass. (right) 0.15 mm thickness of glass.

Table S2. Weight measurement and ratio of PI-glass and LIG-glass composites.

Weight (g)						(LG/PG)
Thickness	Sample No.	Glass	PI-glass(PG)	LIG-glass(LG)	PG-LG	
1 mm	1	1.513	1.566	1.530	0.036	0.977
	2	1.621	1.680	1.640	0.04	0.976
	3	1.803	1.867	1.823	0.044	0.976
0.15 mm	4	0.215	0.268	0.230	0.038	0.858
	5	0.216	0.270	0.232	0.038	0.859
	6	0.214	0.270	0.232	0.038	0.859

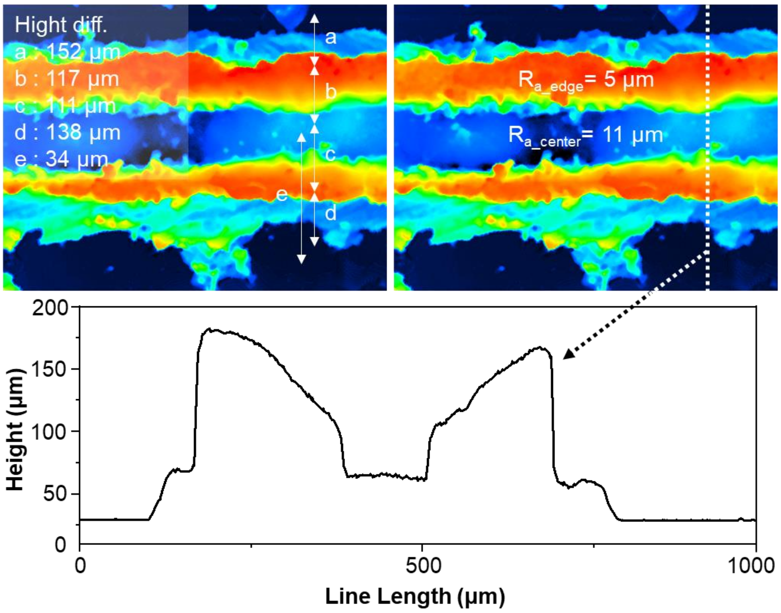

Figure S5. Confocal microscopy results of LIG-glass composites line and cross-section profile of LIG-glass composites.

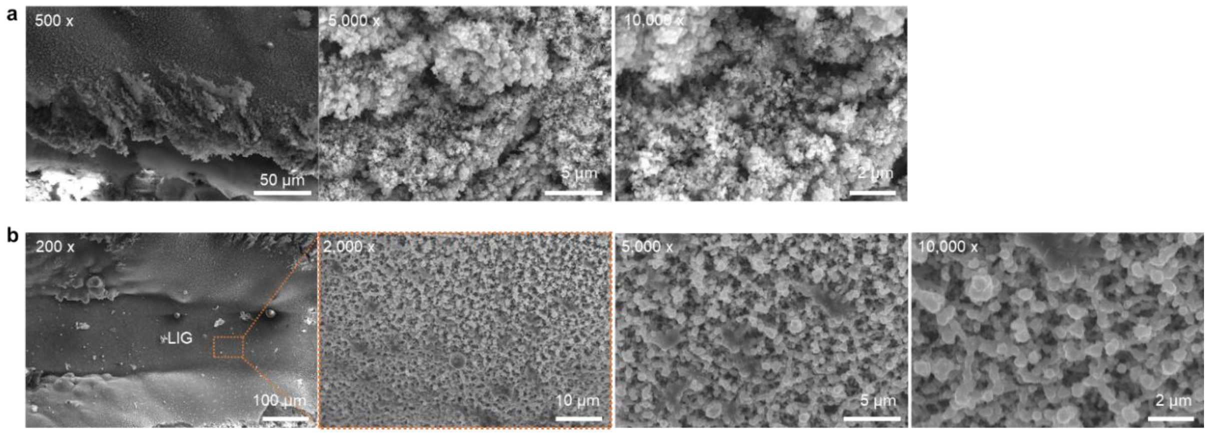

Figure S6. SEM images of LIG-glass composites line in different positions with different magnification.

(a) The SEM parameters for magnifications of
500×: accelerating high voltage (HV) of 20 kV of electron beam, current (curr.) of 0.80 nA, horizontal field width (HFW) of 254 µm, work distance (WD) of 4.5 mm, with Everhart-Thornley detector (ETD).
5000×: 10 kV, 0.40 nA, 25.4 µm, 4.5 mm, with Through-the-Lens Detector (TTLD).
10000×: 10 kV, 0.40 nA, 12.7 µm, 4.5 mm, TLD.
(b) The SEM parameters for magnifications of
200×: 20 kV, 0.80 nA, 635 µm, 4.5 mm, ETD.
2000×: 10 kV, 0.20 nA, 63.5 µm, 4.5 mm, TTLD.
5000×: 10 kV, 0.20 nA, 25.4 µm, 4.5 mm, TTLD.
10000×: 10 kV, 0.20 nA, 12.7 µm, 4.5 mm, TTLD.

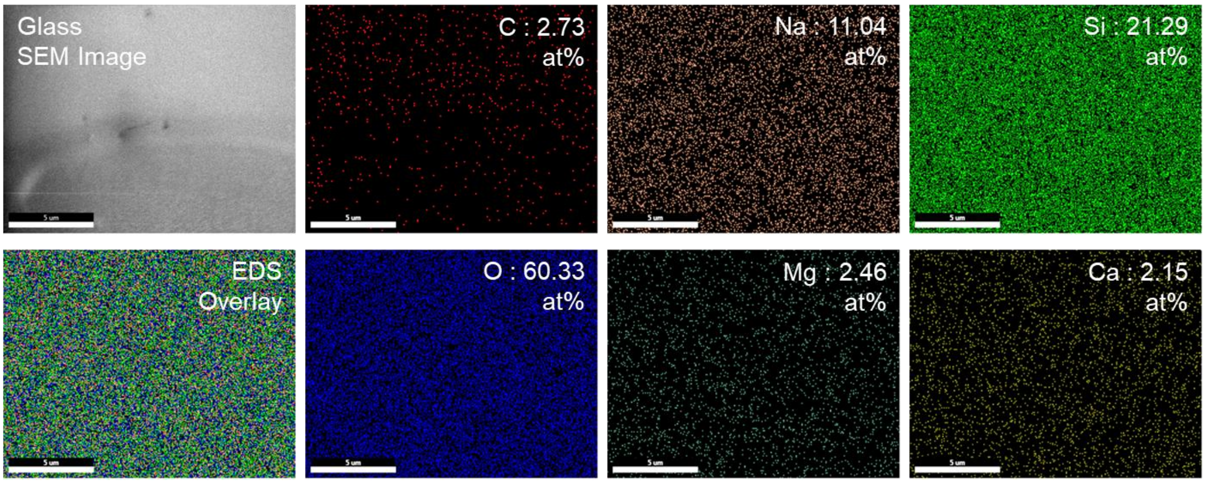

Figure S7. EDS analysis results of glass.

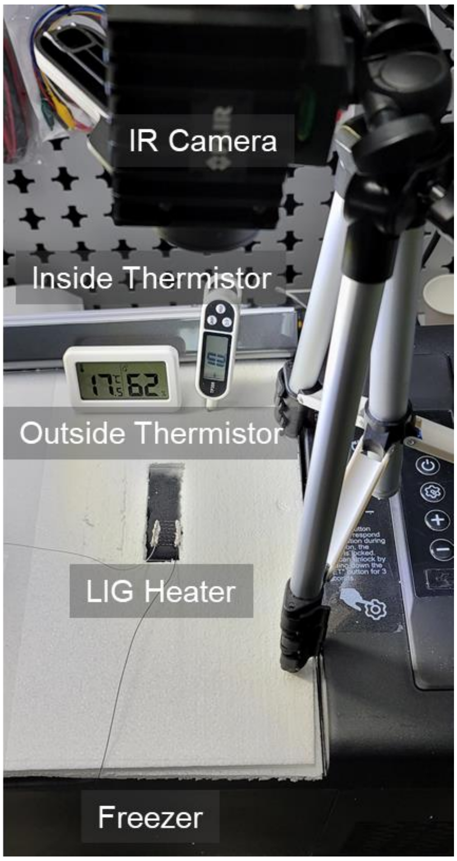

Figure S8. LIG heater set up for defogger test.

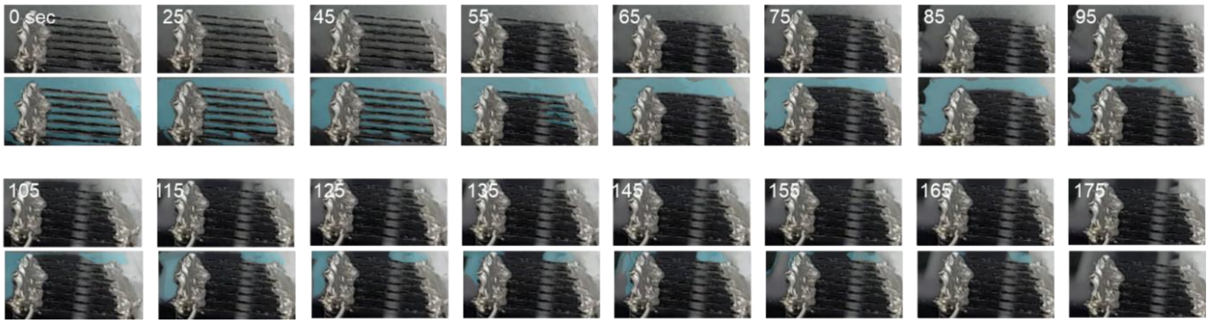

Figure S9. Results of unveiling the fog removal rate over time.

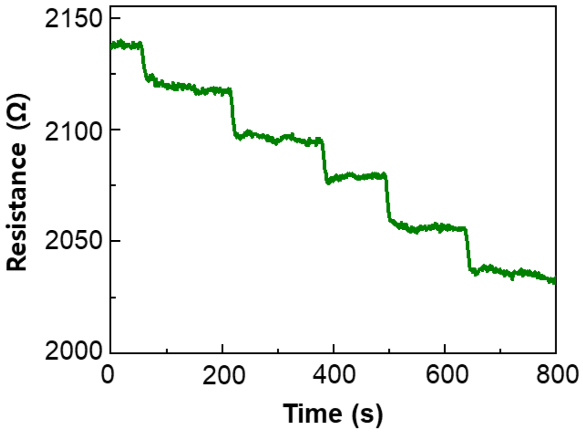

Figure S10. LIG temperature sensor resistance variation with stabilization depending on different temperature.

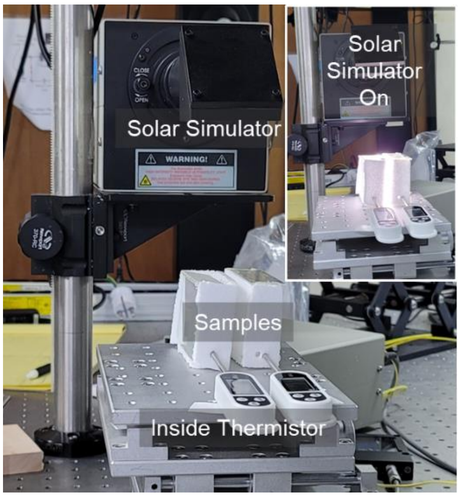

Figure S11. LIG solar warmer set up for monitoring 1D grid solar warmer installed model room temperature.

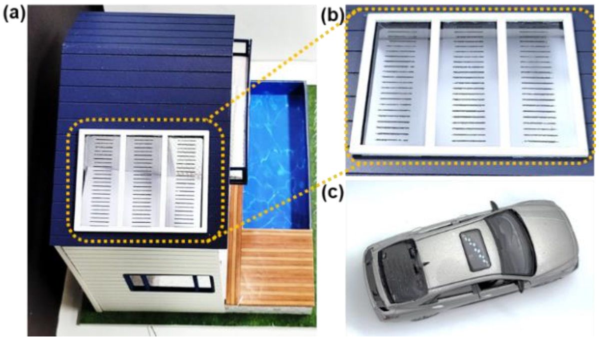

Figure S12. Examples of LIG smart windows installed in house and car windows.

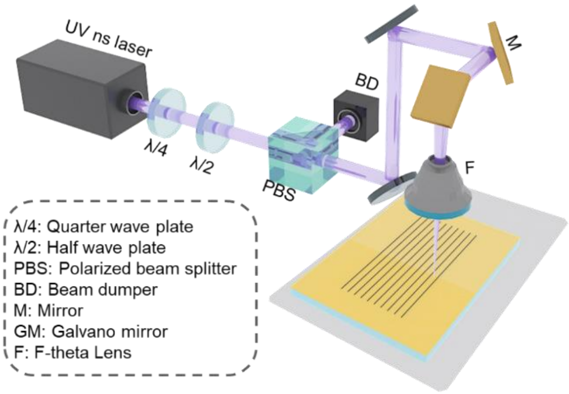

Figure S13. Schematic diagram of the UV-LDW based LIG formation on glass

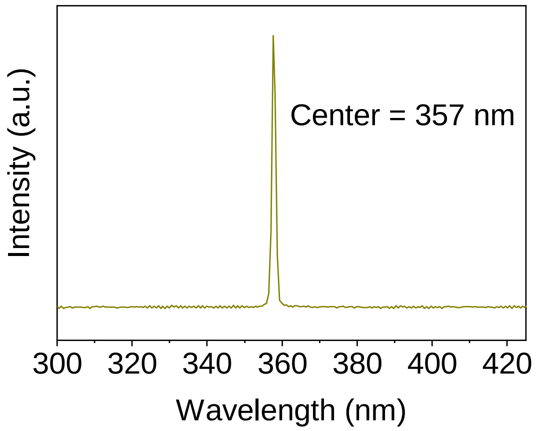

Figure S14. Spectrum of UV nanosecond laser

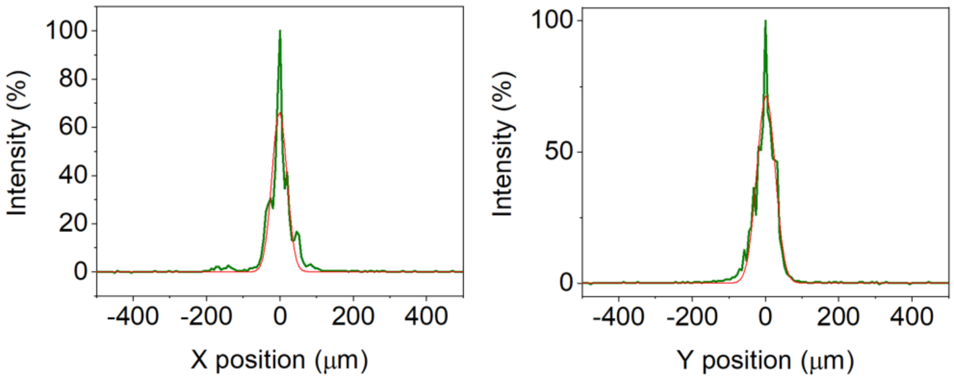

Figure S15. Beam profile of focused beam.

References

[1] Y. Zhou, G. Chen, W. Wang, L. Wei, Q. Zhang, L. Song, X. Fang, *RSC Adv* **2015**, *5*, 79207.
[2] T. Xing, Y. Huang, K. Zhang, J. Wu, *RSC Adv.* **2014**, *4*, 53628.
[3] M. Z. Hlaing, V. Karthikeyan, W. Wu, B. J. Chen, A. K. Ng, C. Chan, M. M. De Souza, V. A. L. Roy, *Adv Opt Mater* **2022**, *10*.
